# Supplementary material for: Are carbon emissions trading and green financial instruments synergistic? -Comprehensive quantitative research based on content analysis
Source: PLoS One. 2024 Mar 7;19(3):e0298601. doi: 10.1371/journal.pone.0298601 (PMC10919601; doi:10.1371/journal.pone.0298601)
Supplement: S1 Data — (ZIP) [file pone.0298601.s001.zip › Data/地级市Z_F工作报告.zip!/╣π╢1⁄2/╜¡├┼╩╨/└┤╫╘╣1⁄2╓┌║┼í╛┬φ┐╦╩2╛▌═°í┐_2014.docx]

各位代表：

　　我代表市人民政府向大会作政府工作报告，请予审议，并请政协各位委员和其他列席人员提出意见。

　　2013年工作回顾

　　过去的一年，市政府在省委、省政府和市委的正确领导下，在市人大及其常委会和市政协的监督支持下，围绕建设幸福侨乡核心任务，狠抓各项工作落实，扎实推进经济社会加快发展和民生改善，全面完成了市十四届人大三次会议确定的目标任务。

　　一、坚持发展第一要务，经济保持平稳较快增长

　　牢牢把握发展第一要务，着力提高经济增长质量效益，有效推动经济工作“稳中有进、稳中向好、稳中提质”。据初步统计，全市生产总值突破2000亿元，比上年增长9.8%；固定资产投资突破1000亿元，增长17.7%；源于江门的财政总收入[1]达到397亿元，其中地方公共财政预算收入158亿元，增长17%；社会消费品零售总额903.7亿元，增长12%;外贸进出口总额197.3亿美元，增长5.1%；金融机构各项存款余额3335.27亿元，增长14.8%，各项贷款余额1715.51亿元，增长16.9%；城镇居民人均可支配收入、农村居民人均纯收入分别增长11%和13%，连续6年实现两位数增幅。

　　大平台建设成效明显。完成大广海湾经济区发展总体规划编制并获省通过。江沙示范园区、鹤山工业城、鹤山龙湾工业园纳入江门国家高新区“一区多园”管理。江门产业转移工业园在全省考核评价中获得第一名。广东轨道交通产业园区、台山工业新城、LED和汽车零部件产业专业园建设进展顺利。江沙食品产业园挂牌。江门国际绿色光源博览交易中心首期封顶。

　　先进制造业优化发展。全市先进制造业增加值增长15%。成功举办江门（上海）投资洽谈会，达成合约77项，投资总额158亿美元。围绕做大做强骨干龙头企业，制定出台诚信企业绿卡计划[2]。广东南车、海信宽带、康师傅顶津项目、普利司通、联成金属、阿博特数码纸业、科隆生物等一批大项目投产。南方电网江门分公司、国华台电、李锦记（无限极）实现主营业务收入超百亿元。天地壹号、维达、富华工程装备制造、冠华针织、中顺纸业、中集建筑模块化、精诚达电路、金牌卫浴等项目增资扩产。成功举办中国（江门）摩托车工业博览会、第五届珠中江进出口商品展销会。

　　科技创新步伐加快。大力推进“四个100”工程[3]，92个项目获省市扶持资金1亿元，带动投资约30亿元。组织实施126项市级以上重点科技计划项目，新培育扶持40家高新技术企业，新增1家国家认定企业技术中心。投入技术创新资金1亿多元，建设6家孵化器，在孵企业超过200家。成为全国科技进步考核先进市和国家知识产权试点城市。全市发明专利申请量增长7%。国家科技型中小企业创业投资引导基金参股我市科技创业风险投资基金。国家机械装备检测重点实验室完成土建工程，建成省质量监督水暖卫浴产品检验站。举办了“五邑杯”科技创新创业大赛、创新工业设计大赛及工业设计活动周，搭建线上工业设计协同创新平台。政企通信息服务平台完成优化升级。中小企业综合服务大厅揭牌运营。

　　现代服务业加快发展。金融对经济发展的支撑作用增强，金融生态环境进一步优化，成为省内首个中小企业信用体系建设实验区。新引进3家银行机构、4家保险机构。侍卫长在“天交所”成功挂牌交易。旅游业蓬勃发展。出台实施了《江门市旅游业发展总体规划（2013－2015）》等一系列扶持办法，成功举办了中国（江门）侨乡华人嘉年华以及台山滨海风情、开平碉楼文化、恩平温泉欢乐、新会葵乡欢乐等旅游节，旅游总收入223.28亿元，增长20.5%。电子商务加快发展，启动实施“江货网店培育工程”。智慧城市建设步伐加快，建成数字城管实景化数据普查等系统，3G网络覆盖全市。

　　二、加快推进基础设施建设，经济发展后劲增强

　　全市固定资产投资完成1000.84亿元，其中重点项目完成投资434.1亿元，投资完成率达108.1%。

　　基础设施建设加快。全市交通建设完成投资49亿元。江顺大桥主塔成功封顶，江门大道北线控制性工程启动施工，西环路隧道贯通在即，广佛江快速通道江门段二期工程列入省重点项目。深茂铁路前期工作扎实推进，广中江高速江门段、江罗高速江门段基本完成征地拆迁，新台高速南延线提前开工建设，中开高速、鹤开高速工程可行性研究评审工作完成，佛开高速陈山出口改造工程完工。成功争取升级两条国道，改写了新会、台山无国道的历史。江门高新区公共码头工程获国家交通运输部批复同意使用岸线，并签订投资框架合作意向书。城乡电网建设完成投资20.3亿元，全社会用电量突破200亿千瓦时。

　　海洋经济加快发展。全市海洋经济实现增加值292亿元，同比增长17%。“海域使用直通车”试点范围从新会区扩大至台山市。广海湾累计填海造地7810亩。全市标准化整治低产鱼塘（咸围）3万亩，崖门国家一级渔港建设竣工，广海国家二级渔港建设立项。新造7艘大功率钢质渔船。全省首个地方海洋协会成立。

　　城镇化扎实推进。启动主体功能区规划编制工作。各市、区加快推进了一批城市道路和综合体建设，如建设路－迎宾路立交、胜利南路、新会启超大道二期、南车路、疏港路一期、江门南站连接线等城市道路通车，新中一级公路、开平环城公路、金瓯路、江睦路、台山台南大道一期等建设扎实推进；万达广场、益丞广场、汇悦城、国际金融大厦、新会美吉特广场、星汇广场、台山中环广场、开平东汇城等城市综合体建设加快。滨江新区全年完成投资8.17亿元、累计完成投资32亿元，启动区路网基本成型，新南路通车，江门体育中心、广东银葵综合医院和范罗冈小学滨江校区建设加快，篁庄考场搬迁工作进入施工阶段。市区新增公交车100.2标台[4]，智能公交系统覆盖公交线路77条，建成候车亭89个。“三旧”[5]改造累计投入104.62亿元，完成改造总面积3230亩，甘化厂片区改造启动。

　　三、扎实推进“三农”工作，城乡发展一体化进程加快

　　农业产业化步伐加快。积极培育新型农业经营主体，培育市级以上重点农业龙头企业58家，新增农民专业合作社777家，农业产业化组织带动农户近40万户，占全市农户的53%。粮食产量创历史新高。开平市“农业科技富民强县专项行动计划”被国家科技部批准立项。全市“三品一标一名牌”[6]农产品总数位居全省前列，成功举办新会陈皮和蓬江杜阮凉瓜文化节，杜阮凉瓜、马冈鹅获国家农产品地理标志认定，台山获评“中国优质丝苗米之乡”。

　　农村生产生活条件明显改善。全市各级财政“三农”投入65.79亿元，比上年增长13.5%。投入民生水利建设5.1亿元，“农田水利万宗工程”[7]和“双千工程”[8]建设扎实推进。落实高标准基本农田建设面积34.65万亩，总投资4.2亿元。落实政策性水稻种植保险面积193.63万亩。投资1亿元完成农村公路建设220公里。农业面源污染治理力度加大。全市完成847条行政村整治规划编制，投入2.9亿元对715条行政村开展村庄整治，创建了一批宜居城镇、宜居村庄（社区）和岭南名镇。新一轮扶贫开发开局良好，80条贫困村累计筹集“双到”[9]资金1.03亿元，实施帮扶项目1600个，已完成885个。积极抓好台风暴雨防御及灾后复产工作。

　　农村管理水平进一步提升。全市所有涉农镇（街）全部建成农村“三资”[10]管理服务中心和交易平台，全年完成农村集体资产资源交易5781宗，成交总金额13.46亿元，高出底价33%。深化“镇财县管”和“村财镇代管”，农村集体财务“组账镇管”制度的覆盖面达98%。新会区成为全国农村综合改革示范试点。

　　四、推进重点领域改革，经济社会发展活力激发

　　经济体制改革取得突破。市属国资整合工作全面铺开，重组成立了市属三大实体公司[11]，为重大基础设施建设和城市运营发展搭建了有效平台。成功发行两只城投债，募集资金超过20亿元。全面开展地方政府性债务审计。举办2013年面向社会投资重大项目推介会，签约项目达15个、投资总额565亿元。民营资本投资活跃，民资完成投资526.59亿元，占全市固定资产投资比重提高至52.6%。全市4952家企业纳入“营改增”试点范围，共计减轻税负8440万元，减税幅度达31.5%。

　　行政体制改革成效明显。推进行政审批制度、简政强镇、事业单位、食品药品监管体制、卫生计生机构等一系列改革并取得明显成效。市一级行政审批项目清理压减事项累计达570项，压减率达43.5%。新成立土地储备、公共资源交易、投资建管等三个中心。进一步理顺市级与蓬江区、江海区两级政府的事权财权关系，做到管理重心下移、权责应放尽放，不断简政强区。实行江海区与高新区合署办公，赋予高新区市一级的管理权限43项，为高新区的加快发展夯实了基础。网上办事大厅实现省、市、县三级网上办事大厅互联互通，全市已进驻网上办事大厅事项共5189项，全部社区（村）设立网上办事大厅自助服务区。市政府门户网站在全国绩效评估中排名前列并获“服务创新奖”。市行政服务中心获全国“人民满意的公务员集体”称号。

　　社会治理改革亮点纷呈。政府购买服务力度不断加大，市本级购买服务项目达到56个、投入资金3486万元，增长124%。全市新建社会组织孵化基地6个，新增社会组织1119个、社工机构18家。全市建成村（居）公共服务站1316个，实现全覆盖，17个街道建有家庭服务中心。全市新增养老床位4954张，比上年增长75%，建设67个农村居家养老服务站，农村养老服务体系建设逐步健全。全市共为1.48万名残疾人提供各类康复服务，顺利通过“全国阳光家园示范区[12]”验收。建成了蓬江和新会星光园居家养老服务中心、恩平利民残疾人服务中心等社会治理创新示范项目。

　　五、加大生态建设力度，宜居环境不断改善

　　园林绿化成效突出。深入开展“森林围城、树林进城”活动，全市投入绿化建设资金4亿元，种树850多万株，参与人次超过70万。新建改建23个大公园和100多个社区公园，新建城市绿道33公里，市区林荫路推广率[13]达88.5%。全市建成区绿化覆盖率43.1%，绿地率41.2%，人均公园绿地面积17.25平方米。完成生态景观林带建设里程208.4公里、碳汇林种植面积3.91万亩、封山育林1.46万亩，新增改造提升森林公园7个，创建森林家园[14]26个。各市、区新建成一批公园如蓬江丰乐山公园、元宝山体育公园一期、金蟾山公园、新会荔枝山公园、石涧公园、南湖公园、中央公园、台山石花山公园、开平人民公园、鹤山公园人文历史雕塑园、恩平锦江湿地公园并免费对市民开放。天沙河两岸景观完成升级改造11公里。台山成为省级园林城市。

　　环境保护力度加大。高度重视潭江综合整治工作，制定出台工业、农业、生活污染整治工作方案。大力度、大手笔解决污水处理设施历史欠帐多的遗留问题，全市污水处理设施建设投入3亿元，比上年增长118%，为近年来投入力度最大的一年。建成棠下污水厂首期、高新区污水厂首期、江海污水处理厂技术改造扩建工程，新建污水管网74公里，新增污水处理能力8万吨/日，全市生活污水处理率达到84%。加大环境执法力度，全市立案查处环境违法案件654宗，停产整治环境违法企业113家，移送公安机关涉嫌环境污染犯罪案件10宗。荷塘镇环保专项整治行动成效显著。全市城镇生活垃圾无害化处理率达96.2%，建成镇级生活垃圾转运站57座，全市11852个自然村设有生活垃圾收集点，实现自然村全覆盖。实施中心城区“黄标车”[15]限行，淘汰“黄标车”7574辆。

　　节能减排和节约集约用地水平提升。在全国率先推行排污权有偿使用和交易。完成重点减排项目107项。实施省级节能改造项目5个，实现节能量近万吨标准煤。完成市区路灯LED改造1.2万盏。银洲湖纸业基地、崖门环保电镀基地等循环经济模式加快发展。出台政策鼓励旧厂房升级改造，严把项目用地准入门槛，加大闲置土地处置力度，全市盘活存量土地超万亩。连续13年实现耕地占补平衡，获得省耕地保护综合考评一等奖。江门市及恩平、鹤山获得全省土地执法监察考核奖励。新会区被评为全国国土资源节约集约模范区。

　　六、大力发展文化事业，侨乡文化得到保护与传承

　　以“五邑银信”[16]为重要组成部分的《侨批档案》入选世界记忆遗产名录。启动实施“四馆”[17]整合建设工程。五邑图书馆及新会、台山、开平、鹤山、恩平等市（区）图书馆被评为国家一级图书馆，江门市博物馆被评为国家二级馆。江门甘化厂工业遗产保护工作有效推进。成功举办梁启超诞辰140周年纪念活动、陈白沙文化节、中国（江门）工艺美术精品文化博览会暨台山玉雕刻创作大赛、江门侨乡动漫节、南粤幸福活动周、社会科学普及周等活动。全年新建（改造）城乡社区文化室308个，建成文化信息资源共享工程基层服务点200个，农村放映电影13600场，送戏下乡650场。实现广播电视“渔船通”[18]。

　　七、千方百计保障改善民生，人民群众生活水平进一步提高

　　全市各级财政民生投入98亿元，增长18.2%，十件民生实事全面完成。“创文”成果进一步巩固，长效机制逐步健全。开平市、台山市“创文”工作通过省的测评，恩平市启动创建工作。据国家统计局广东调查总队测评报告显示，我市群众幸福感总体评价名列全省第二。

　　就业形势稳定。建立了就业实名制信息系统和“一对一”就业帮扶机制[19]。全市城镇新增就业4.71万人，失业人员再就业3.42万人，城镇登记失业率2.32%。全市开展农村劳动力技能培训2.61万人，新增转移农村劳动力3.1万人。全市培养高技能人才1.56万人。

　　社保覆盖面进一步扩大。全市城镇职工基本养老、基本医疗、失业、工伤、生育保险参保人数分别比上年增长1.18%、1.14%、6.04%、8.4%、7.59%。提高企业退休人员养老金、失业保险金、最低工资标准。完善全征地农民养老保障政策，保障力度和待遇水平大幅提高，受惠群众2.76万人。大力推进社会保障“一卡通”，全市八成户籍人口共327万人实现“人手一卡”。城乡低保标准进一步提高，五保[20]集中供养、分散供养标准分别比上年提高33%和20%。全面落实高龄老人津贴发放工作。超额完成省下达的保障房建设任务，新开工保障性住房3141套，基本建成4781套。华侨农场危房改造有效推进，新开工207套，基本建成222套。

　　社会事业不断进步。全面启动创建省教育现代化先进市工作，“固强创强”工作扎实推进。全市幼儿学前入园率达到96%，义务教育阶段公办学校达标率为100%。高考再创佳绩，本科入围率创历史新高。全市积分制入户农民工子女入读义务教育阶段公办学校达100%。“平价医院、平价诊室、平价药包”建设扎实开展。建成一批国家、省和市卫生镇村。市妇幼保健院新院建成并投入使用。完成农村卫生改厕1万户任务。妥善处置各类突发公共卫生事件，有效防控H7N9禽流感、登革热等疫情。全民健身深入农村，建成乡镇全民健身广场17个，社区健身设施覆盖率达到90%。人口计生工作完成省下达目标任务。国防动员工作取得明显成效。成功举办慈善公益万人行和扶贫济困日活动，共筹善款近1.6亿元。妥善处置“9?29”渔船遇险事件善后工作。此外，侨务、外事、港澳、对台、民族、宗教、双拥、优抚、人防、统计、妇女儿童、方志、气象、红十字会等工作进一步加强。

　　平安江门建设扎实推进。建成市区公安智能交通控制中心项目。社区警务建设深入推进，科技强警力度加大，有效保持平安江门品牌。全面开展超标电动车和校车安全专项整治。社会信用体系和市场监管体系建设深入推进，全市餐饮食品、保健食品和药品生产经营单位安全信用档案建档数达8090家，实现全覆盖。推进安全生产标准化建设，安全生产隐患治理成效显著，各项安全生产事故指标再创新低。市本级投入2636万元用于战勤保障中心建设、购买高层云梯车和无人机等先进装备，消防应急救援能力进一步提升。落实信访维稳领导“一岗双责”和包案责任制，有效解决一批涉及群众切身利益的问题，确保社会大局稳定。

　　八、政府自身建设不断加强，依法行政和服务水平有效提升

　　依法行政水平进一步提升，我市被评为全国法治城市、法治县（市、区）创建活动先进单位。自觉接受市人大及其常委会的监督，充分利用人大代表约见市长、政协委员约见市长和提案直通车等制度，多渠道听取人大代表、政协委员意见，抓好建议提案办理工作，人大议案和建议、政协提案办理满意率均达100%。成立重大决策专家咨询论证委员会，开通市政府政务微博，坚持民生热线制度，科学民主决策水平进一步提高。严格贯彻落实中央“八项规定”，坚决抵制“四风”[21]，政府作风不断改善。“文山会海”大幅削减，市政府印发公文和召开会议同比减少46%和29%。严控“三公”经费[22]支出，网上公开“三公”经费，主动接受社会监督。市本级压减5%公用经费用于民生支出。严格控制楼堂馆所建设，全面清查违规办公用房用车现象。加强廉政风险防控，推进廉洁城市建设，坚决查处违纪违法案件，始终保持惩治腐败高压态势。

　　各位代表，过去一年政府工作取得的成绩来之不易，这离不开上级党委、政府和市委的正确领导，离不开市人大、市政协与各民主党派的监督支持，离不开全市人民、外来投资者和建设者以及驻江门军警部队的共同努力，离不开港澳台同胞、海外侨胞以及国际友人的关心帮助。在此，我代表市人民政府，向所有参与、支持和关心江门发展建设的各界人士，表示衷心的感谢！

　　与此同时，我们也清醒地看到经济社会发展中还存在不少突出问题：投资结构性矛盾突出，实体经济投资比重下降；自主创新驱动力不足；城乡基础设施规划建设和社会公共服务有待继续完善；政府服务水平和办事效率有待进一步提高。这些问题，我们将高度重视，采取有力措施加以解决。

　　2014年工作安排

　　2014年是贯彻落实十八届三中全会精神的第一年，也是完成“十二五”规划目标任务的重要一年。尽管面临的国内外经济形势仍然复杂多变，但做好今年的工作还是有很多有利条件，特别是我市经济发展保持稳中向好的趋势，投资拉动作用进一步加大，金融对经济发展的支撑力显著增强，城镇化和第三产业繁荣推动，加上中央、省对全面深化改革作出了重大部署，必将释放出新的动力和活力。今年政府工作的指导思想是：深入贯彻落实党的十八届三中全会、中央经济工作会议、省委十一届三次全会和市委十二届四次全会精神，抓住改革这一主题和发展这一主线，坚持稳中求进总基调，坚持发展实体经济不动摇，继续以“种树、搭桥修路、抓大项目”为抓手引领全局工作开展，努力推动幸福侨乡建设再上新台阶。

　　今年全市经济社会发展的主要预期目标是：生产总值增长10%，人均生产总值增长9%；地方公共财政预算收入增长11.5%；固定资产投资增长18%，社会消费品零售总额增长13%，外贸进出口总额增长4%；城乡居民人均收入增长11％，居民消费价格涨幅控制在3.5%以内，城镇登记失业率控制在3.2%以内；节能减排约束性指标完成省下达的目标任务。

　　为实现上述目标，必须做好以下工作：

　　一、加强大项目大平台建设

　　今年安排市重点项目210项，年度计划投资451亿元，其中安排现代产业项目97项，年度计划投资164.7亿元；能源保障项目6项，年度计划投资120亿元；产业园区建设项目11项，年度计划投资33.5亿元，着力强化投资特别是实体经济投资的拉动作用。

　　扶持骨干龙头企业和优势产业加快发展。推进实施诚信企业绿卡计划，加大力度扶持壮大一批产值超10亿元的骨干龙头企业，带动实施“133”优势产业提质增量工程，即大力培育1个产值超1000亿元产业、3个超300亿元产业和3个超200亿元产业。抓好高端新型电子信息、轨道交通装备制造、生物医药、新光源、新能源、新材料等战略性新兴产业发展，推动机电、纺织、造纸、食品、建材等传统产业加快转型升级、扩能增效，提高产业竞争力。办好中国（江门）绿色（半导体）光源国际博览会。

　　加快重大基础设施建设。进一步完善全市“内通外联”的交通网络，对接广佛都市圈、港珠澳大桥，实现江顺大桥主跨合龙，西环路隧道全线贯通，加快江门大道北线、深茂铁路、江罗高速、广中江高速、新台高速南延线、江门高新区公共码头建设进度，抓紧推进江门大道南线、广佛江快速通道江门段二期工程、广佛江珠城轨、鹤开高速、中开高速、佛开高速三堡至水口段扩建前期工作。

　　推进大广海湾经济区规划建设。按照“五个定位”[23]的要求，抓好《大广海湾经济区发展总体规划》的实施，加快专项规划编制，完善管理架构和运营体制，推进起步区规划建设。完成市、县两级海洋功能区划修编工作，启动大广海湾区集中集约用海规划和区域用岛规划编制工作，完成广海湾区填海造地新增1000亩。加大省市共建海洋经济强市力度，大力推进“海域使用直通车”试点工作。加快崖门万吨级航道、广海湾港区进港航道整治工程前期工作。加快现代渔业发展，抓好广海国家二级渔港建设，加快推进横山渔港申报国家一级渔港和沙堤渔港申报国家中心渔港工作。

　　二、狠抓招商引资

　　加大项目引进和增资扩产力度。制定实施招商引资工作行动纲要和三年行动计划。加强全产业链招商，注重引进价值链高端产业。进一步创新招商方式方法，完善招商机制，整合招商资源，走出去到国内经济发达的大城市举办投资洽谈会。积极引入外企、央企、民企500强来我市进行战略布点，选择我市中小企业兼并重组和产业配套。抓好2012年江门市招商推介会、2013年广东江门（上海）招商洽谈会以及面向社会投资重大项目推介会签约项目及投资线索的跟进工作，建立健全项目落户协调服务机制，加快项目落户建设速度。鼓励发展总部经济。抓好全市现有优质企业增资扩产，加快推进各市、区26个、总投资超200亿元的增资扩产项目建设。

　　提升核心工业园区建设水平。按照现代企业制度要求创新园区建设运营的理念，通过资源整合、平台融资、股权投资等手段，突破资金瓶颈，重点抓好基础配套设施建设，打造高水平投资载体。加快推动江门高新区实施“一区多园”和“新三板”扩容工作。加快广东轨道交通产业园基础设施建设，力争引进核心配套企业落户。加大力度建设江沙示范园区、银洲湖纸业基地、崖门环保电镀基地、台山工业新城、鹤山工业城等园区。抓住省推进产业园区扩能增效契机，抓好江门产业转移工业园扩园和新产业转移园申报设立工作，推动全市工业园区建设上规模、上档次。

　　转变外贸发展方式。稳定进出口规模，培育出口竞争新优势。大力推动加工贸易转型升级，促进跨境电子商务和外贸新业态发展，打造以“江门制造”为核心的区域品牌形象。办好第六届珠中江进出口商品展销会，积极开拓内销市场。继续深化口岸联检部门合作，进一步简化报关报检手续，大力推进“三个一”[24]口岸通关模式，深化通关作业无纸化改革，切实提高通关效率。加快出口退税进度。

　　三、推进产业结构转型升级

　　强化创新驱动。加强科技招商，健全完善产学研协同创新机制，积极引进重大科技成果和吸纳高层次科技领军人才，密切跟进中关村科技交流对接后续项目。推动科技和金融的紧密结合，推进以“三资融合”[25]模式建设高新区总部科技园，加快科技孵化器、服务平台等科技创新载体建设。促进专业镇公共创新平台建设。充分发挥工业设计公共服务平台作用，推动工业设计与制造业深度结合。加快国家机械装备检测重点实验室建设。推进江门市技术交易中心建设。组织实施100项市级重点科技计划项目，组织开展产学研项目80项，推广科技成果500项，创建市级以上企业工程研究开发中心10家。全面推进质量强市战略，鼓励企业争创名牌和参与国际、国家、行业标准制（修）订，推动高新技术企业开展产品认证申报工作。

　　培育发展民营经济和中小企业。毫不动摇地鼓励、支持、引导非公有制经济发展，激发民营经济活力。重点围绕中小企业占主导的传统产业集群，积极搭建营销公共服务平台、产品质量检验检测平台、出口认证公共服务平台，充分发挥商会行业协会作用，加强中小企业综合服务体系建设。进一步构建完善中小企业融资平台，大力推介金融创新产品，鼓励中小微企业在全国中小企业股份转让系统、天津股权交易所和香港H股挂牌融资，推进高新区试点知识产权质押融资。全面落实“营改增”政策，进一步清理现有行政事业性收费，切实减轻企业负担。深化帮扶建筑业企业发展，进一步振兴建筑业。抓好第三次全国经济普查。

　　加快现代服务业发展。进一步提高金融支撑实体经济发展的能力，推进中小企业信用体系实验区建设，加大金融产品和服务创新力度。进一步把旅游业作为支柱产业来打造，加强旅游项目招商，大力统筹整合全市旅游资源，打造生态文化、滨海度假、休闲侨都三大旅游产业集聚区，加快推进开平马降龙古村落、碉楼小镇、新会陈皮村、银湖湾游艇休闲度假区、希尔顿逸林酒店、台山颐和温泉城、黄花梨博物馆、传统家具文化展示街、恩平恒大泉都旅游城等项目开发建设。进一步发展生产性服务业，推广制造业主辅分离、全程供应链一体化管理等现代化物流模式，实施中小物流企业信息化提升工程。进一步推动城市经济繁荣发展，加快万达广场、保利城市综合体、新会美吉特商业综合体、星汇广场、台山英皇广场、嘉旺城国际公馆、开平东汇城、恩平锦江新城等城市综合体建设，加强商业网点规划和公益性农产品批发市场建设，推进万村千乡市场工程[26]升级改造和农超对接。加快培育信息消费、医疗保健、养老服务、家庭服务、体育健身、教育培训等新兴消费性服务业。推进工艺美术、文化用品、印刷出版、文化创意设计、演艺娱乐等文化产业优化升级。

　　四、全面深化改革

　　深化经济体制改革。引入竞争机制优化公共资源配置，发挥三个新组建运营中心[27]作用，加强公共资源交易能力建设和管理体制建设。深化国资整合发展工作，不断提升国有企业的发展质量和运行效率，继续做好江门融浩水业股份有限公司上市的前期培育工作。深化财税体制改革，预算管理试行全口径编报，建立完善预算编制征询和审查监督机制。继续推进市直财政资金绩效评价和行政事业单位财政资金绩效管理考评。加强市级专项资金规范管理，提高资金使用效益。扩大政府购买服务范围和规模，制定出台指导目录，完善政府向社会转移职能制度。推进市财政经营性资金实施股权投资管理改革。加强地方政府性债务管理，逐步建立地方政府性债务风险预警机制和偿债准备金机制。进一步落实市对蓬江区、高新区及江海区财政体制改革方案。深化台山、开平、鹤山三家信用联社改革，争取年底达到改制农村商业银行的基本条件。鼓励、引导民间资本设立民营银行、村镇银行、小额贷款公司、融资担保公司等机构。继续放宽主体准入、投资准入条件，鼓励民间投资参与基础设施、城建、水利、社会养老等公共领域，积极发展混合型经济。

　　深化行政体制改革。进一步推进简政放权，健全完善行政审批事项目录管理和公示听证制度。重点围绕促进企业投资和项目建设，逐步有序、最大限度减少行政审批事项。做好新一轮市县政府职能转变和机构改革工作。继续深化事业单位改革。按照“宽进严管”原则大力推进商事登记制度改革，进一步降低市场准入门槛。着力完善网上办事大厅建设，推进政务公开专栏建设，简化网上办事流程，年底90%以上的行政审批事项实现网上受理，进一步优化营商环境，使得政府服务更便利、更优质、更高效、更贴心。

　　深化社会治理体系改革。加快社会组织的培育发展，进一步提升社会组织参与社会治理的能力。大力支持和发展志愿服务组织，推进志愿者和社工的联动。大力推动社工人才培养，加强社会工作发展平台建设，力争全市每个城镇社区设置1－2个社工岗位，鼓励有条件的农村社区设置1个社工岗位。推进社区管理精细化、信息化，开展社区网格化管理试点工作。创新有效预防和化解社会矛盾体制，积极推动司法行政基层“三进”[28]工作转型升级，努力构建覆盖城乡的公共法律服务体系。

　　五、推动农业大市向农业强市迈进

　　抓好现代农业发展。加快农业园区建设，推进开平国家现代农业示范区、粤台（江门）农业合作试验区和台山都斛万亩水稻高产示范田建设。培育壮大农民合作组织、农业产业化龙头企业、家庭农场、种养大户等新型经营主体,新增省、市级农业龙头企业2家和4家，农民专业合作社达1200家，农户入社率达30%。开展市级农业综合改革试点镇创建工作。加快发展政策性农业保险，探索建立农业投融资平台，实施农业“政银保”[29]项目。完善扩展涉农财政补贴政策信息服务功能，全面推广应用“江门市农村信息直通车”服务平台。切实保障粮食安全。实施优质粮工程，大力发展提升园艺产业、冬种农业和禽畜标准化规模养殖。改造提升地方特色农业，推动“三品一标一名牌”认证。办好第四届农业博览会、第三届中国（江门）锦鲤博览会。

　　加强农村基础设施建设。全面完成34.65万亩和启动新建29.98万亩高标准基本农田建设任务。加强农田水利设施建设，加快推进山塘及病险水库除险加固、千里海堤达标加固和中小河流治理工程建设。完成150公里农村公路建设任务。继续实施世界银行贷款农业面源污染治理项目，注重农业生态环境保护，加强土壤治理，突出抓好禽畜养殖污染减排，实现养殖废弃物资源利用率80%以上。抓好“美丽乡村”工程，继续开展行政村整治规划编制工作，新增63条村庄开展整治工作、累计占全市行政村总数的74%。继续抓好宜居城镇、宜居村庄和幸福村居、名村示范村创建工作，建成3个宜居城镇、14个宜居村庄、10个名村示范村。继续实施村级公益事业建设“一事一议”项目。积极探索新一轮扶贫开发长效机制，加快80条贫困村帮扶项目建设，确保扶贫“双到”工作取得实效。

　　强化农村管理。继续深化农村综合改革，积极开展农村土地承包经营权登记试点工作。抓好新会区国家农村综合改革示范试点实施工作。推进农村集体“三资”管理制度化、规范化、信息化，保障农民集体经济组织成员权利。切实抓好村级组织换届选举工作。

　　六、提高城乡宜居水平

　　促进城市扩容提质。发挥规划引领作用，推进规划“一张图”[30]工作，进一步提高城市控规覆盖率。积极推进主体功能区规划，科学谋划产城融合，促进新型工业化和新型城镇化互动协调发展。加快推进市区73项、年度投资43.1亿元的城市建设项目进度。全面推进滨江新区建设，抓好启动区二期开发，制定新区配套产业规划，加快江门体育中心、广东银葵综合医院、滨江新区中小学建设。积极推进珠三角城际轨道交通江门南站与滨江新区TOD[31]项目规划建设。稳步推进“三旧”改造，加快甘化厂片区、江会路片区升级改造，同时保护好历史文化街区。各市、区要依托城关镇和中心镇引导小城镇专业化、集约化、特色化发展，建设一批侨乡味道浓、文化底蕴深、生态环境美的风情小镇，提升城镇化水平。

　　开展新一轮绿化大行动。深入开展“森林四进”[32]、“园林下乡”及节日种树活动，广泛发动群众和鼓励引导单位、社区、企业厂区种树绿化。完善城市绿化格局规划，进一步完善绿道配套设施。逐步开展立体绿化，每个市（区）推进1－2条试点街（路）。规划建设江门植物园。加强林相改造工作，年内建设生态景观林带39.2公里、碳汇工程林4.56万亩。每个市（区）建设1－2个市级生态园林示范镇、10－15条市级生态园林示范村。

　　加大环境保护力度。坚持把生态文明建设摆在更加突出的位置，保护好江门五邑的自然生态和山山水水。加大潭江流域水环境综合整治力度，完善组织协调机制，严格落实责任制，深入开展工业、农业、生活污染治理，形成有效监督机制。加大污水处理厂及配套管网建设力度，加快建设荷塘污水厂、杜阮污水厂、恩平污水处理厂二期扩建工程、新会7个非中心镇污水处理厂和各市、区污水管网，全市城镇污水处理率达87%。加大大气污染防治力度，继续推动“黄标车”淘汰，加强建筑工地管理，抓好粉尘危害治理，推进全市环境空气质量监控网建设，做好PM2.5等污染物的监测工作。加大城乡生活垃圾处理力度，全面推广垃圾分类试点工作，城镇生活垃圾无害化处理率达96.3%，做好农村生活垃圾处理处置，实现全市城乡垃圾收集、运输、处理全覆盖。强化饮用水源保护，推进江门市区应急备用水源建设。全面加强重金属污染防治。加快推进江门市再生资源环保处置中心筹建工作。加强噪音扰民的管理。建立健全企业环境保护管理制度，完善环境保护行政执法与刑事司法衔接机制，依法严惩破坏环境违法行为。

　　促进资源节约集约利用。落实最严格的耕地保护制度。积极推进节约集约用地，严格执行工业项目用地准入条件，加大盘活存量土地力度，全市盘活存量土地不少于8000亩。完善节能减排联动机制，强化节能减排责任考核。加快循环经济发展，继续淘汰落后产能。大力发展绿色节能建筑。加大推广应用LED照明产品力度，积极开展市区LED路灯节能改造二期项目建设。

　　七、促进以改善民生为重点的社会各项事业发展

　　今年各级财政预算民生投入108亿元，比上年增长20.1%。继续承诺为人民群众办好十件民生实事，实现发展成果更多更公平惠及市民群众。

　　推进“大民政”建设。重点围绕提升养老服务和残疾人保障等底线民生水平，构建完善对困难群体、特殊群体和优抚群体的救助制度和优惠政策。推进养老服务体系建设，打造各市、区居家养老示范中心，完成市福利院养老楼主体工程建设和敬老院升级改造试点项目，促进“医养结合”的养老模式，进一步探索推进区域性养老。按照不低于省调整的新标准发放重度残疾人护理补贴和低保残疾人生活津贴，大力推进残疾人居家康复、居家养护等居家服务，扶持残疾人发展生产及自主创业。抓好社会保险扩面征缴工作，完善完全被征土地农民养老保障工作机制。提高全市医疗救助标准，引入市场机制提高大病保障水平。继续提高低保救助水平和五保供养水平。积极打造侨乡慈善品牌，探索推行市、县（区）、镇（街）三级慈善体系建设，组织好慈善公益万人行和扶贫济困日活动。抓好保障性住房建设和华侨危房改造工作。

　　实施就业优先。实现全市城镇新增就业4.5万人以上，城镇失业人员再就业3万人，应届高校毕业生就业率达90%以上。加强创业孵化基地建设，加大对各类创业人员的扶持力度。继续落实“一对一”就业帮扶工作，重点深化特殊群体就业服务工作。完善就业实名制系统，进一步提高人力资源市场服务水平。不断强化劳动监察保障工作，全市创建和谐劳动关系示范区工程的企业参与率达到60%。

　　加快教育改革发展。进一步巩固“创强”成果，持续推进教育现代化建设。推进普惠性幼儿园建设，创建9所以上省一级幼儿园。统筹城乡义务教育资源配置，促进普通中小学校高位均衡优质发展。推进“名师工程”，加强高素质专业化教师队伍建设。重视国学教育，深入推进中华经典诵读活动。继续改善五邑大学和江门职业技术学院办学条件。积极筹办江门幼儿师范高等专科学校。继续完善现代职业教育体系，实施中等职业教育改革发展示范学校建设工程，积极探索组建职业技术教育集团，大力推进实训中心和市级职业技术教育基地建设。加快建立健全终身教育体系。发挥引进高层次人才“一站式”服务平台作用，优化人才发展环境。创新多元教育机制，完善市教育发展基金，出台鼓励社会力量办学的实施意见，大力发展民办教育。进一步完善外来工子女受教育权保障制度和助学政策体系。

　　推动文化惠民。进一步完善公共文化服务设施，推进“四馆”整合建设和江门大剧院前期工作。加强公共数字文化建设，继续推进数字图书馆工程、公共电子阅览室、网上博物馆建设。加强全市历史文化建筑的调查与保护，做好长堤风貌街、甘化厂工业遗产的保护和开发利用。推进新会陈皮、台山大江传统家具制造技艺申报国家级非物质文化遗产名录工作。继续实施农村电影放映工程，深入开展文化下乡、进社区活动。办好侨乡阅读文化节暨动漫节。开展中国特色社会主义和“中国梦”宣传教育活动，培育和践行社会主义核心价值观，大力弘扬华侨精神。进一步健全和落实“创文”长效机制，抓好“创文”迎检工作。

　　保障群众健康。深化医药卫生体制改革，进一步优化配置全市医疗资源，开展县（市）公立医院综合改革试点，支持和引导民营医疗机构发展，提升全市医疗质量和竞争力。逐步建立社区卫生服务机构与公立医院上下联动、分工协作机制，加快社区首诊、分级诊疗制度的建立。持续抓好急、重大传染病和艾滋病防控。继续抓好平安医院建设，加快建立医患纠纷调解委员会体制。积极开展爱国卫生运动，争创省市卫生镇2个。改造农村卫生户厕1万户。确保完成省下达的人口计生任务。推进全民健身深入城乡社区，积极推动公共体育场馆及有条件的学校体育场地对外开放。

　　建设便捷出行交通。坚持公交优先，持续加大公交车购置改造、公交候车亭建设等公共交通投入，加快公交首末站、枢纽站的规划建设。抓好市区道路、高速公路出入口的建设改造以及重要地段、重点路口的改造升级，统筹安排1.8亿元财政资金推进市区沥青路改造和城市道路微循环系统[33]等项目建设。加快推进北环路、福泉路、迎宾西路、杜阮北一路、胜利南路延长线、篁庄大道西延线、金瓯路、连海北路、新会新峰路二期、台山台南大道、开平环城公路、鹤山中山路延伸段、恩平环城快速路一期等市政道路建设改造。推进出租车经营权投放。抓好公共停车场的规划、建设和管理。

　　建设平安江门。深入推进“平安江门”十大工程[34]建设。抓好立体化社会治安防控体系建设，严打暴力犯罪、多发性侵财犯罪，大力推动禁毒斗争，继续推进治安重点地区整治。健全落实网上舆情引导处置机制，强化信息网络安全管理。深入推进社区警务建设，提升基层基础工作水平。推动信访制度改革，畅通群众诉求表达、利益协调、权益保障渠道，做好综治信访维稳工作。深化安全生产管理体制改革，严格落实“党政同责、一岗双责、齐抓共管”，强化安全生产基层基础建设和企业主体责任落实，坚决遏制较大以上事故发生。建设和完善基层食品药品监管体系，强化食品药品安全监管。加快完善山体消防通道和设施，成立专业森林消防队，抓好森林防火。完善公共突发事件应急处置体系建设，提高应急管理能力。做好国防动员与优抚安置工作，巩固“双拥”模范城创建成果。

　　此外，继续加强外事、侨务、港澳工作，开好第四届世界江门青年大会，扎实推进华侨农场改革发展和侨青创业园建设。继续做好民族、宗教、对台、人防、打私、统计、审计、档案、方志、气象、妇女儿童、红十字会等工作。

　　八、加强政府自身建设

　　建设为民务实清廉政府是时代的要求、人民的期盼。要始终牢记宗旨，勤勉尽职，继续大力提升执行力、协同力、公信力和创新力，建设法治政府和服务型政府。

　　推进依法行政。运用法治思维加强政府建设，抓好依法治市工作。认真执行市人大及其常委会各项决议决定，主动接受人大监督，鼓励并支持政协委员参政议政，提高人大代表议案、建议和政协委员提案办理质量。健全完善各项制度，用制度约束权力，用制度监督权力。进一步规范行政自由裁量权行使，逐步完善程序立规，强化常态化监督。改革行政执法体制，推进综合执法、统一执法，促进运用法律手段、依靠司法程序解决有关执法问题。不断加强绩效考核、行政问责，扎实推进政府绩效管理电子监察制度建设。

　　推进作风转变。坚持严格执行中央“八项规定”，大力开展党的群众路线教育实践活动，突出解决机关“四风”问题。进一步完善政企通服务平台建设，依托“12345”政府服务热线开通政企通服务热线，力争年底覆盖2万家企业。进一步健全行政复议、投诉申诉、网络问政等利民便民制度，畅通民意表达渠道。推动政府信息公开化，创新信息发布机制，健全完善重大事项听证程序，扩大公众对政府工作的有序参与。大力推动重大行政决策专家咨询论证工作，保证依法决策、民主决策、科学决策。

　　推进廉政建设。抓好廉洁城市建设，严格落实党风廉政建设责任制。加强公务用车管理，实现“三公”经费和人员编制只减不增，稳步推进预决算和“三公”经费信息公开工作。强化对政府性资金和投资项目的审计监督。加强惩治商业贿赂长效机制建设。严肃查处违法违纪案件，营造风清气正、勤政高效的政务环境。

　　各位代表！做好今年的各项工作有赖于大家的支持理解和全市人民的共同努力，让我们在省委、省政府和市委的正确领导下，继续解放思想，改革创新，凝心聚力，攻坚克难，为建设幸福侨乡作出新的贡献！
